# *Plagiorchis muris* (Tanabe, 1922) in *Rattus norvegicus* in Iran

**Published:** 2013

**Authors:** Gholamreza MOWLAVI, Iraj MOBEDI, Hoda ABEDKHOJASTEH, Seyed Mahmoud SADJJADI, Farideh SHAHBAZI, Jafar MASSOUD

**Affiliations:** 1 Dept. of Parasitology and Mycology, School of Public Health, Tehran University of Medical Sciences, Tehran, Iran; 2 Center for Research of Endemic Parasites of Iran (CREPI), Tehran University of Medical Sciences, Tehran, Iran; 3 Dept. of Parasitology and Mycology, School of Medicine, Shiraz University of Medical Sciences, Shiraz, Iran

**Keywords:** *Plagiorchis muris*, *Rattus norvegicus*, Trematode, Zoonoses, Iran

## Abstract

Rats are capable to harbor various pathogens, among which certain species of zoonotic parasites are included. A long-term detection of parasite fauna of rats has sporadically been carried out in Iran. Abundance of these vertebrate pests is of great importance as regards public health issue. The present paper is focused on a digenean trematode *Plagiorchis muris*, obtained during a comprehensive study on rats over the decades in the country. Herein we describe this occurrence in a *Rattus norvegicus* in northern Tehran, with specific note on its morphological description. *P. muris* can infect human through consumption of infected marine food items, and has never been observed in Iran.

## Introduction

Various parasitic helminthes among rodents including rats; *Rattus rattus* and *R. norvegicus*, have been reported from all over the world (1, 2). Biological behavior of these opportunistic small mammals has facilitated the acquiring and developing of some zoonotic agents as well as specific parasites (2, 3). Merely two exclusive parasites are well known in rats: *Trichosomoides crasicuda*, commonly named as “Rat bladder worm” and the most prevalent blood protozoa *Trypanosoma lewisi*. However, many researchers have reported faunistic surveys on rat parasites throughout the world (1, 4, 5) and to some extent in Iran (6, 7) as well. For instance, parasites of rodents such as *Plagiorchis muris* has been studied in *Apodemus silvaticus* in Spain (8).

To our knowledge, *P. muris* has not been recorded in rodents in Iran yet. Several of mammals harbor this intestinal fluke, which feeds freshwater snails such as Lymnaeids as the first intermediate host (9). *Bllamya bengalensis*, also has recently displayed to be infected with xiphidiocercariae of Plagiorchiidea (10). Mean-while, aquatic insects, insect naiads and fresh water fishes are playing as second intermediate host (11). Furthermore, like other trematodes, *P. muris* is appearing in a place with multiple environmental and biological factors. Phylogenetic studies indicated that certain species belonging to suborder Plagiorchiata have a paraphyletic nature and parasitize only terrestrial vertebrates (12). Natural human infection however has been reported from Korea, in which, freshwater fishes, snails and insects were claimed as the source of infection among the residents in riversides (9).

The present paper has illustrated the occurrence of *P. muris* in an urban pest, *R. norvegicus* which unexpectedly found infected in Tehran, Iran.

## Case report

From 2000 to 2010 in a sequential rat controlling program in Iran, random sampling were carried out in the capital Tehran, Ahwaz in the southwest, and Bandar Anzali on the Caspian littoral. Five hundred collected rats were sacrificed individually using diethyl ether anesthesia in the cage or were found dead during the environmental monitoring. Dissection was carefully performed while internal organs were precisely investigated for harboring helminthes. Digestive tract was split lengthwise and gut contents were observed routinely by stereomicroscope. Attained trematodes were prepared to identify morphologically and stained by carmine acid. Complete morphologic and morphometric characters leading to reliable identification were analyzed using a camera lucida. To have a comparative observation, characteristic features of the worm were photographed microscopically and identified taxonomically, based on references (13, 14).The rest of the parasites recovered through these occasions have been exclusive to the rodents and are not regarded in this paper. Out of 500 dissected rats, only one *R. norvegicus* was seen naturally infected by *P. muris* with a worm burden of sixty-four. Of those numbers, 19 individuals with appropriate structural condition were selected for measuring and illustrating of morphological characters.

Characters including length 2891 – 1618 (2167) µm, width 831 – 509 (689) µm, distance between the middle points of oral and ventral suckers 765-375 (514) µm, middle of ventral sucker to the posterior end 2151 – 1033 (1473) µm, proximal eggs to the genital pore 41-33.6 × 21.5-15.5(38.4×17.4) µm were carefully measured.

**Fig. 1:**
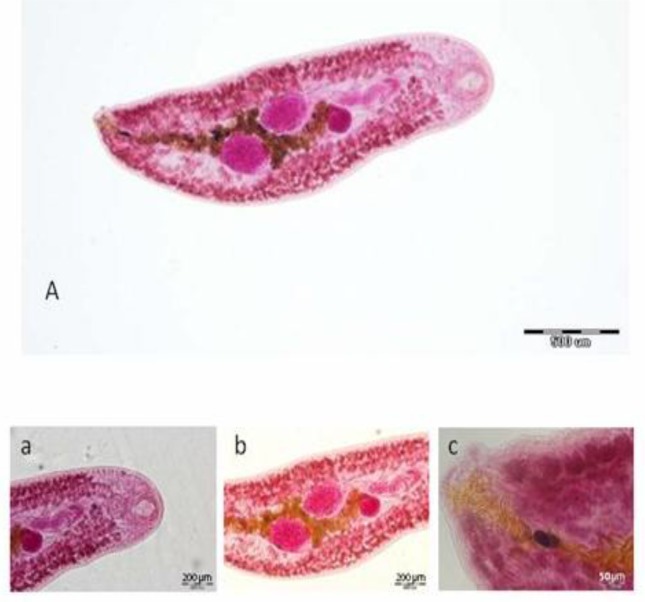
*Plagiorchis muris* removed from natural infected *R. norvegicus*, (A) whole body. (a) Anterior parts: oral sucker, pharynx and ventral sucker, (b) middle part: ovary, testis and parts of uterus, (c) posterior end

**Fig. 2:**
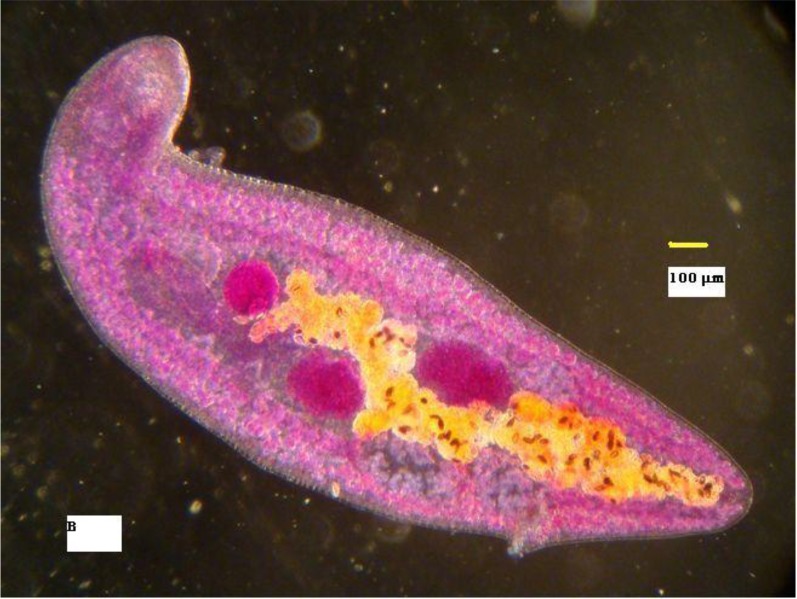
Whole body stained with Carmine acid, using phase contrast microscopy

Morphological and morphometrical characters were found compatible with those *P. muris* that have been already documented in key references (14). The entire body, anterior parts (oral sucker, pharynx and ventral sucker), middle part (ovary, testis and parts of uterus), and posterior end are illustrated below (Fig. 1,a–c, Fig. 2).

**Fig. 3:**
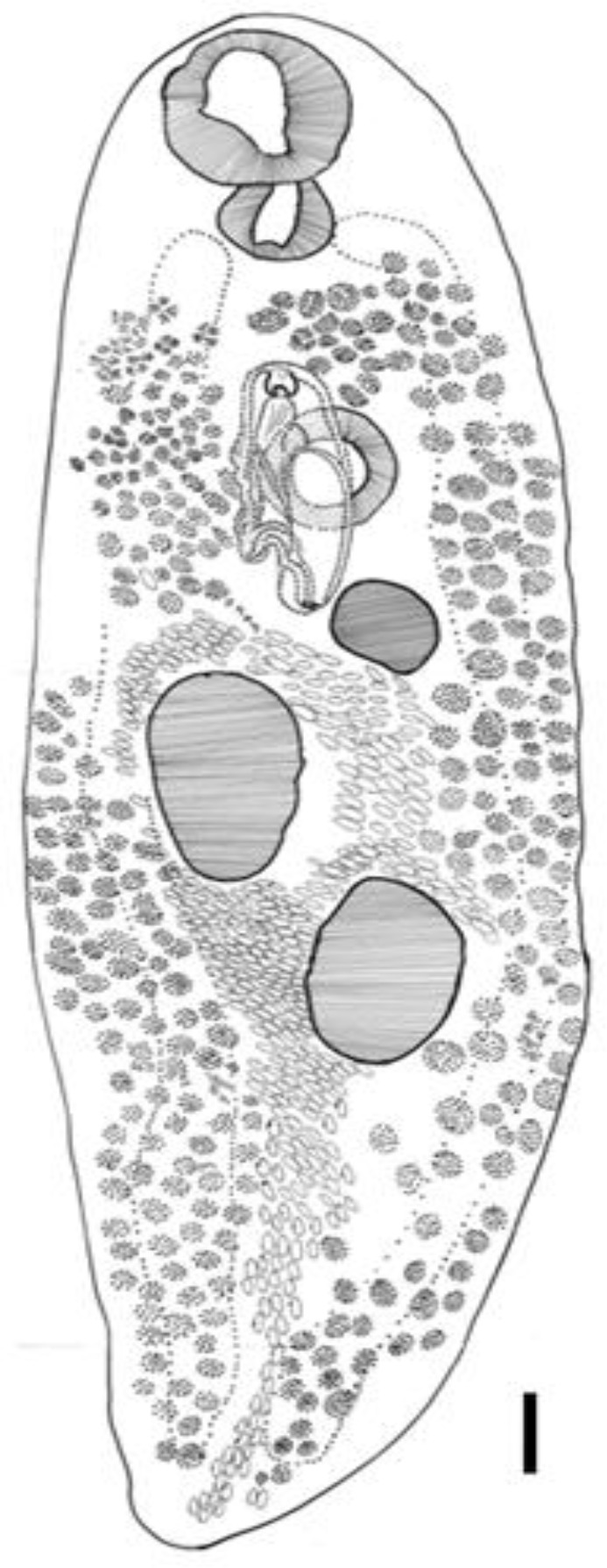
*Plagiorchis muris* in brown rat *(Rattus norvegicus)* of Tehran, Iran a schematic drawned by Camera Lucida (Scale bar= 100 µm)

The fluke morphology in details illustrating herein has drawn by the use of camera lucida (Fig. 3).

## Discussion

Comparing with many other prevalent helminthes in rodents, *P. muris* is not rather common one, which can be browsed throughout the literature (4, 15, 16). *P. muris* was described for the first time in *R. rattus* and *R. norvegicus* in Japan (17). Since then it has been reported from different hosts such as *R. rattus*, *Crocidura* sp and *Apodemus agrarius* in Taiwan, India and Korea respectively (18–20). Parameters measured for these recovered helminthes, were seen similar in size range with those afore-mentioned references.

The rarity of this tiny trematode among considerable number of examined rats, agrees with indication of life cycle complexity that should necessarily employs two biological intermediate hosts in a same foci (21). From public health perspective, *P. muris* occurrence can be of concern in populations with tendency to seafood and exotic foods, carrying metacercariae as edible items incidentally. Cases that may occur under an unusual condition are exemplary in Japan and Korea (9, 22, 23).

Concerning the human transmission, the expectation seems to be low in Iran due to the cultural behavior, which does not support the means of transmission. Appropriate eco-biological conditions, which had been established for this case in a limited urban place with good level of hygiene in Tehran, attract the researchers to investigate on the life cycle of *P. muris* in the given area.

The absence of *P. muris* in the list of the several surveys conducted so far on rat parasitic fauna (6, 7) can be attributed to digenean life cycle complexity. To evaluate the present environmental potentials, which might be led to transmit this zoonotic helminth to humans, a comprehensive field study is needed to be performed in crowded cities of the country.
